# Effects of premedication with oral gabapentin on intraocular pressure changes following tracheal intubation in clinically normal dogs

**DOI:** 10.1186/s12917-017-1206-1

**Published:** 2017-09-19

**Authors:** Alexandra Trbolova, Masoud Selk Ghaffari, Igor Capik

**Affiliations:** 1 Clinic of Small Animals, University of Veterinary Medicine and Pharmacy, Kosice, Slovakia; 2 Department of Clinical Sciences, College of Veterinary Medicine, Karaj Branch, Islamic Azad University, Alborz, Iran

**Keywords:** Gabapentin, IOP, tracheal intubation, dogs

## Abstract

**Background:**

Gabapentin is an antiepileptic drug widely approved as an add-on therapy for epilepsy treatment in human and dogs. There is a clinical impression that gabapentin is a suitable drug which attenuates the IOP elevation associated with tracheal intubation in humans. The present study performed to determine the effects of oral gabapentin on intraocular pressure (IOP) changes following tracheal intubation in dogs.

**Results:**

Twenty adult healthy dogs were randomly assigned to treatment (*n* = 10) and control (*n* = 10) groups. Dogs in the treatment group received oral gabapentin (50 mg/kg) 2 h before induction of anesthesia and dogs in the control group received oral gelatin capsule placebo at the same time. The dogs were anesthetized with propofol 6 mg/kg, and anesthesia was maintained with a constant infusion of 0.2 mg/kg/min of propofol for 20 min. IOP were measured immediately before induction and then repeated immediately after induction, as well as 5 min, 10 min and 15 min following tracheal intubation in both groups. IOP was significantly higher immediately after induction, and 5 min after tracheal intubation when compared with IOP reading before induction in the control group. There was no statistically significant change in IOPs immediately after induction, and 5 min after tracheal intubation in comparison to the values before induction in the treatment group.

**Conclusions:**

Based on the findings of this study, preanesthetic oral administration of gabapentin significantly prevents an increase in the IOP associated with tracheal intubation in dogs anesthetized with propofol.

## Background

The sensitivity of maintaining an intraocular pressure within or below reference range throughout sedation or general anesthesia has been the subject of extensive research in humans and animals [1–3].

An appropriate anesthetic protocol for any patient with an ophthalmic problem should include not only drug selection but also a preoperative management plan to provide an optimal postoperative outcome [4]. Abrupt increases in intraocular pressure associated with anesthetic drugs or orotracheal intubation can cause dramatic effects in patients with near-perforating corneal lesions or glaucoma [5]. Hofmeister et al. reported a significant increase in IOP after intubation compared to before induction with propofol in dogs [1].

Gabapentin is an antiepileptic drug widely approved as an add-on therapy for epilepsy treatment in human and dogs [6–9]. There is a clinical impression that gabapentin is a suitable drug which attenuates the IOP elevation associated with tracheal intubation in humans [10].

As far as we know there is no published work on the effects of gabapentin on IOP following orotracheal intubation in dogs. The aim of this study was to determine the effects of oral gabapentin on IOP changes following tracheal intubation in dogs.

## Methods

This study was done with agreement of the Ethic Committee (protocol number: **2048/02–17.**) of the University of Veterinary Medicine and Pharmacy, Kosice, Slovakia, in accordance with Slovakian ethical codes for studies on laboratory animals (All dogs were research animals).

Prior to entering the study, all dogs were determined to be free of disease by means of physical and ocular examination using biomicroscopy, tonometry and indirect ophthalmoscopy. No obvious abnormalities were noted during the ophthalmic examinations.

Twenty adult healthy dogs were included in this study. The dogs were fasted overnight but had free access to water. Dogs were randomly assigned to treatment (*n* = 10) and control (*n* = 10) groups. Dogs in the treatment group received oral gabapentin (50 mg/kg; Teva Pharmaceuticals CR, S.R.O. Praha, Czech Republic; the gabapentin was compounded in order to assure an accurate dose) 2 h before induction of anesthesia and dogs in the control group received oral gelatin capsule placebo at the same time.

After 5 min of preoxygenation, the dogs were anesthetized with propofol (6 mg/kg IV; Fresenius Kabi, GmbH, Graz, Austria; entire calculated dose of propofol was given), and anesthesia was maintained with a constant infusion of 0.2 mg/kg/min of propofol for 20 min.

IOPs were measured immediately before induction (baseline; Tb) and then repeated immediately after induction (T0), as well as 5 min (T5), 10 min (T10) and 15 min (T15) following tracheal intubation in both groups. For all dogs, the same investigator (A.T.) performed all IOP measurements. (A.T.) obtaining IOP blinded to treatment group assignment. During tonometry, the animals were placed in sternal recumbency with head in normal and upright position and the eyelids were not manipulated as measurements were taken.

Before the tonometry, a drop of proparacine hydrochloride (Paracain, Sunways Ltd., Mumbai, India) was applied in both eyes of the dogs immediately. IOP was measured using applanation tonometry with the Tono-Pen AVIA VET (Tono-Pen AVIA, Reichert, NY). The tonometer was factory calibrated before initiation of the study. Only IOP readings with a 5% variance (5% displays on Tono-Pen; we recorded IOP values once we got that one reading with a 5% variance) were recorded.

Cardiorespiratory monitoring was performed by means of assessing hemoglobin saturation (SpO_2_), pulse rate, respiration rate, and systolic blood pressure at T_b_, T_0_, T_5_,T_10_, T_15_ using Mindray- PM - 9000 Vet (Mindray, Hamburg, Germany).

Statistical analysis was performed by using the software package SPSS version 15.0 for Microsoft Windows (SPSS Inc., Chicago, Illinois 60,606, USA). Data were expressed as mean ± SD. Shapiro-Wilk test was used to evaluate whether the data were normally distributed. A 2-way ANOVA was used to compare data within the same group and to assess differences between groups. A *p*-value of <0.05 was considered statistically significant.

## Results

**Fig. 1:**
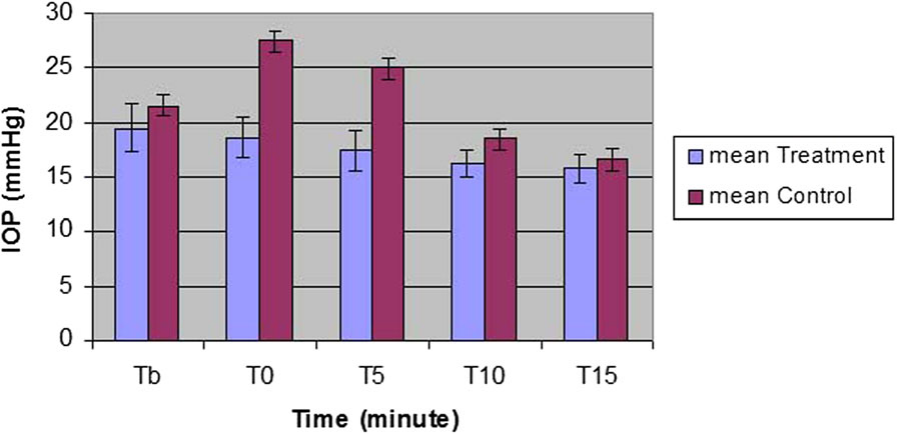
Mean and SD of intraocular pressure in treatment (10 dogs, received oral gabapentin 2 h before induction of anesthesia) and control (10 dogs, received oral gelatin capsule placebo at the same time) groups at baseline, 0, 5, 10 and 15 min after induction of anesthesia

**Fig. 2:**
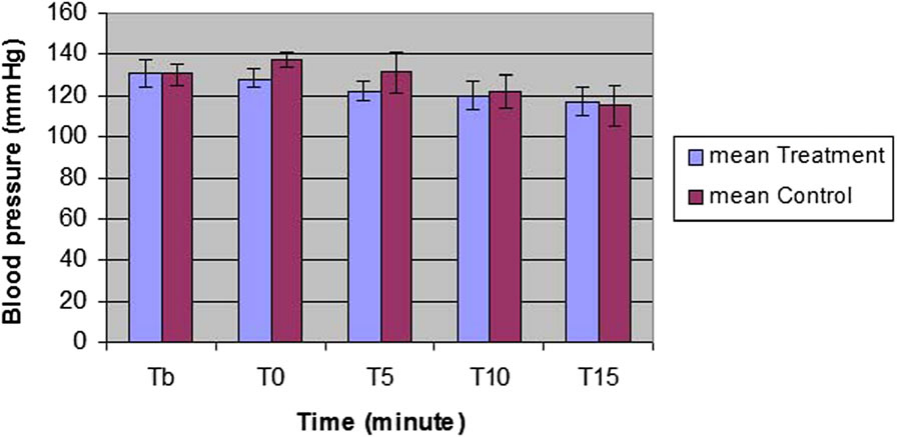
Mean and SD of blood pressure in both treatment and control groups at baseline, 0, 5, 10 and 15 min after induction of anesthesia

The mean values and SD of IOP and blood pressure in both treatment and control groups are shown in Figs. 1 and 2. All data are expressed as mmHg.

The mean ± SD baseline IOP values (obtained immediately before induction; Tb) for treatment and control groups were 19.5 ± 2.2 and 21.6 ± 2.6, respectively. For baseline IOP values, there was no significant difference between treatment and control groups (*P* = 0.05).

IOP was significantly higher immediately after induction (27.5 ± 3.1; P_0_ < 001), and 5 min after tracheal intubation (25.0 ± 2.4; P_5_ = 0.001) when compared with IOP reading before induction (21.6 ± 2.6) in control group. The systolic blood pressure showed no statistically significant increase after induction (137.5 ± 4.1; P_0_ = 1.00), also recording values at 5 min after tracheal (131.9 ± 9.4; P_5_ = 1.00) was still higher than values at before induction (130.6 ± 4.9) but it was not statistically significant.

Comparison between treatment IOP values in the control group revealed significant differences between T0 and T5 (*P* = 0.004) as well as comparison between T5 and T10 (*P* = 0.00).

There was no statistically significant change in IOPs immediately after induction (18.6 ± 1.9; P_0_ = 0.9), and 5 min after tracheal intubation (17.4 ± 1.8; *P* = 0.6) in comparison to the values before induction (19.5 ± 2.2) in the treatment group. Comparison between treatment IOP values in the treatment group revealed no significant differences (*P* ≥ 0.9).

There is a tendency for a systolic blood pressure decreasing effect in treatment group when comparing values with recorded values prior to induction, The mean ± SD values before induction, immediately after induction and at 5 min after tracheal intubation were 131.0 ± 6.3, 128.5 ± 4.0 (*P* = 0.9), and 122.3 ± 4.6 (*P* = 0.2) respectively.

In both groups, the recorded values for IOP and systolic blood pressure at 15 min after intubation showed statistically significant decrease (*P* < 0.05).

## Discussion

This study evaluated the effects of oral gabapentin on IOP changes following tracheal intubation in dogs. At clinically useful doses (10–60 mg/kg), maximum blood levels, reached within 1–3 h after peroral administration [11, 12].

This information helps to explain our results in which oral gabapentin significantly prevent the increase in IOP associated with endotracheal intubation. These findings were similar to a previous study in humans which documented gabapentin as a useful adjuvant in order to prevent an increase in the IOP in response to tracheal intubation [10].

The baseline values of IOP observed prior to treatment in the dogs presented in this study were similar to those reported in normal dogs [13, 14].

The exact mode of action of gabapentin in decreasing IOP in dogs is unclear. Several mechanisms have been proposed to explain gabapentin modulation of IOP in humans. Intraocular blood volume is determined by arterial inflow, venous outflow, and tone of the intraocular vasculature. These variations in intraocular blood volume modify the IOP [4]. It could be suggested that the possible relaxing effects of gabapentin on the ciliary muscle by inhibiting membrane voltage-gated calcium channels, (acting in a manner similar to calcium channel blockers) might lead to the attenuation of the IOP by improving the flow of aqueous humour through the canal of Schlemm [10, 15].

In the present study, post-treatment IOP measurements were compared with baseline and negative control group IOPs. In this investigation, the control group was considered to allow proper evaluation the effects of time, anesthesia, and intubation as variables on IOP readings.

An interesting finding in this study was a significant effect of gabapentin on blood pressure**.** In the present work, we found a statistically significant decrease in systolic blood pressure 5 min after intubation, when compared with baseline values.

Our findings are in agreement with previous studies in humans which showed that gabapentin attenuated blood pressure increase following tracheal intubation by its inhibitory effects on membrane voltage-gated calcium channels [15, 16].

In fact, the success of an ophthalmic surgery may depend on control of IOP before, during and after the procedure. Anesthetic management should minimize changes, specifically an increase in IOP over the entire anesthetic period.

Maintaining an IOP within or below reference range throughout general anesthesia is essential for certain ocular diseases. For example, corneal lesions that approach full thickness require delicate handling and control of IOP to avoid inadvertent perforation prior to and during surgery. Corneal perforations, particularly those that result in iris prolapse, have a notably worse prognosis for preservation of vision postoperatively [17, 18]. Therefore; exploring safer treatment for abrupt increase of IOP in dogs undergoing ocular surgery is warranted.

## Conclusions

Based on the findings of this study, preanesthetic oral administration of gabapentin significantly prevents an increase in the IOP associated with tracheal intubation in dogs anesthetized with propofol. As gabapentin appears to be useful drug in attenuation of increased IOP associated with intubation, its administration can be reliably advised when performing ocular procedures in dogs with deep ulcer or laceration in which increased IOP may result in ocular complications, such as perforation of the globe.

## Abbreviations

IOP: Intraocular pressure
